# *VAV3* Oncogene Expression in Colorectal Cancer: Clinical Aspects and Functional Characterization

**DOI:** 10.1038/srep09360

**Published:** 2015-03-20

**Authors:** Yih-Huei Uen, Chia-Lang Fang, You-Cheng Hseu, Pei-Chun Shen, Hsin-Ling Yang, Kuo-Shan Wen, Shih-Ting Hung, Lu-Hai Wang, Kai-Yuan Lin

**Affiliations:** 1 Department of Medical Research, Chi Mei Medical Center, Tainan, Taiwan; 2 The Superintendent's Office, Chi Mei Hospital Chiali, Tainan, Taiwan; 3 Department of Pathology, School of Medicine, College of Medicine, Taipei Medical University, Taipei, Taiwan; 4 Department of Pathology, Wan Fang Hospital, Taipei Medical University, Taipei, Taiwan; 5 Department of Cosmeceutics, China Medical University, Taichung, Taiwan; 6 Department of Molecular and Cellular Oncology, The University of Texas MD Anderson Cancer Center, Texas, USA; 7 Department of Health and Nutrition Biotechnology, Asia University, Taichung, Taiwan; 8 Institute of Nutrition, China Medical University, Taichung, Taiwan; 9 Department of Pharmacy, Chi Mei Medical Center, Tainan, Taiwan; 10 Institute of Molecular and Genomic Medicine, National Health Research Institutes, Miaoli, Taiwan; 11 Department of Nutrition, Chia Nan University of Pharmacy and Science, Tainan, Taiwan

## Abstract

Although colorectal cancer (CRC) is one of the most common malignancies worldwide, the current therapeutic approaches for advanced CRC are ineffective. In this study, we investigated the involvement of the *VAV3* oncogene in tumor progression and in the prognosis of human CRC. The two patient cohorts in this study comprised 354 CRC cases from 1998 to 2005 with documented pathologic and clinical factors and clinical outcomes. VAV3 protein levels were significantly correlated with the depth of invasion (*P* = 0.0259), the nodal status (*P* < 0.0001), distant metastasis (*P* = 0.0354), the stage (*P* < 0.0001), and poor disease-free survival (*P* = 0.003). Multivariate Cox regression analysis showed that VAV3 overexpression is an independent prognostic marker for CRC (*P* = 0.041). In vitro experiments indicated that VAV3 knockdown inhibited CRC cell growth, spread, and xenograft proliferation. Mechanistic studies further revealed that VAV3 overexpression could dysregulate the expression of cell cycle control- and metastasis-related molecules by activating the PI3K-AKT signaling pathway in both CRC cells and xenografts. This study suggests that VAV3 overexpression could be a useful marker for predicting the outcomes of CRC patients and that VAV3 targeting represents a potential modality for treating CRC.

Colorectal cancer (CRC) accounted for over 1.2 million new cases of cancer in 2008 (9.4% of the global total)^1^,^2^. In Taiwan, CRC ranks as the most frequently diagnosed malignancy and causes more than 4900 deaths annually (http://www.doh.gov.tw/statistic/index.htm; accessed in December 2013). Although the current surgical techniques and chemotherapy have significantly improved, the cure rate for advanced CRC remains low and the morbidity remains high^3^. Thus, advances in the treatment of this disease are likely to come from a better understanding of its pathogenesis and biological features. Many studies have suggested the role of genetic alterations in the development and progression of CRC^4^,^5^. Molecular markers might be helpful not only to understand the disease pathogenesis but also to provide a useful prognosis.

VAV3, a GEF for Rho family GTPases, belongs to the VAV protein family^6^. The VAV proteins contain multiple functional domains and are involved in various cellular signaling processes, including regulating cytoskeleton organization, cell transformation, and oncogenesis^7^,^8^. Receptor protein-tyrosine kinases in various signal transduction pathways directly or indirectly activate VAV proteins. VAV3, a downstream signal transducer of EGFR/HER2, has been shown to bind to several partners, including PI3K, leading to cell transformation, including alterations in cell morphology^9^. VAV3 overexpression leads to PI3K activation and focus formation in NIH3T3 cells, and blocking PI3K activity by LY294002 efficiently inhibits VAV3-induced cell transformation^10^. However, the roles and underlying mechanisms of VAV3 overexpression in cancer cell growth and spreading are not well understood.

Research related to the prognostic value of VAV proteins is limited. VAV1 overexpression is an independent prognostic marker for pancreatic cancer^11^. Gene amplification and protein overexpression of VAV3 appear in several types of human cancer, including breast cancer, glioblastoma, and prostate cancer^12^,^13^,^14^. It was recently shown that VAV3 could serve as a marker of recurrence and survival for patients following prostatectomy of early stage cancers^15^. Our previous study was the only one to show that VAV3 overexpression is an independent prognostic marker for gastric cancer^16^. To the best of our knowledge, the expression and prognostic significance of VAV3 in CRC remains unknown.

In the present study, we conducted immunohistochemical analysis of VAV3 expression in 354 primary CRC specimens to examine its clinical significance in CRC and analyzed its possible association with the clinicopathologic parameters of the tumors, as well as with patient survival. We then performed small hairpin RNA (shRNA)-mediated gene silencing to investigate the effect of VAV3 on the biological behavior of CRC cells and discussed the possible mechanisms involved in the genesis and metastasis of CRC.

## Results

### VAV3 expression was up-regulated and associated with several clinicopathologic parameters in CRC

**Figure 1:**
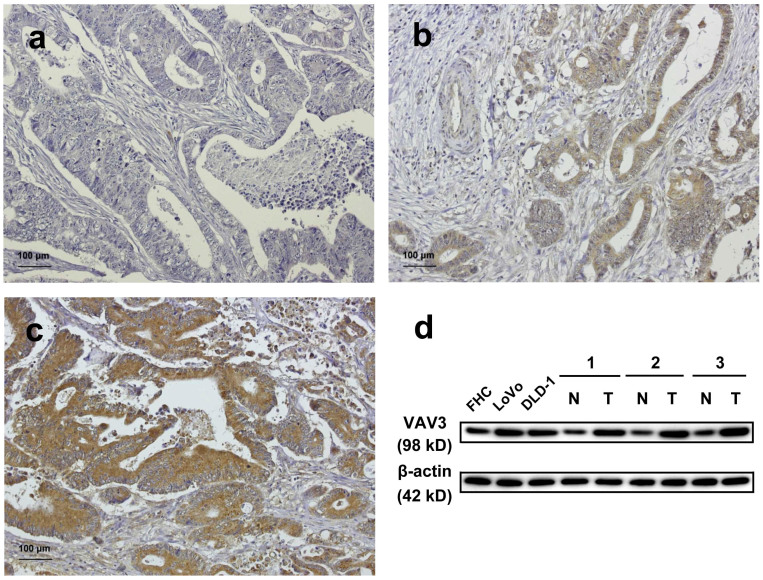
VAV3 expression in colorectal tissues and cell lines. (a–c) CRC analyzed by immunohistochemistry with an antibody against VAV3. Panel a shows a sample without VAV3 expression; Panel b shows a sample with low VAV3 expression; Panel c shows a sample with high VAV3 expression. (d) Endogenous VAV3 protein expression was remarkably increased in CRC cell lines (LoVo and DLD-1) and tissues. N and T stand for non-tumor and tumor, respectively. No. 1, 2, 3 were patients number. The blots in the figure were cropped, but the polyacrylamide gels were run under the same experimental conditions.

This study immunohistochemically investigated the expression of VAV3 in two patient cohorts. VAV3 expression was higher in tumor tissues than in non-tumor tissues in both data sets (*P* < 0.001). Eleven percent of the tumors in data set two were totally negative (score 0), and 39% were weak and focal staining in <25% of tissues (score 1) (Figure 1a and 1b). Data set two also revealed a high expression or overexpression of VAV3 in 50% of tumors (33% with a score of 2 and 17% with a score 3, Figure 1c). Immunoblotting also revealed that VAV3 expression was higher in CRC cells (LoVo and DLD-1) and tissues than in normal cells (FHC) and tissues (Figure 1d). Supplementary Table S1 summarizes the clinical features of these two cohorts of patients. In data set one, VAV3 overexpression was significantly correlated with the depth of invasion (*P* = 0.0266), nodal status (*P* < 0.0001), and stage (*P* < 0.0001). In data set two, VAV3 overexpression was significantly correlated with the depth of invasion (*P* = 0.0259), nodal status (*P* < 0.0001), distant metastasis (*P* = 0.0354), and stage (*P* < 0.0001).

### VAV3 overexpression and patient survival in data set one

**Figure 2:**
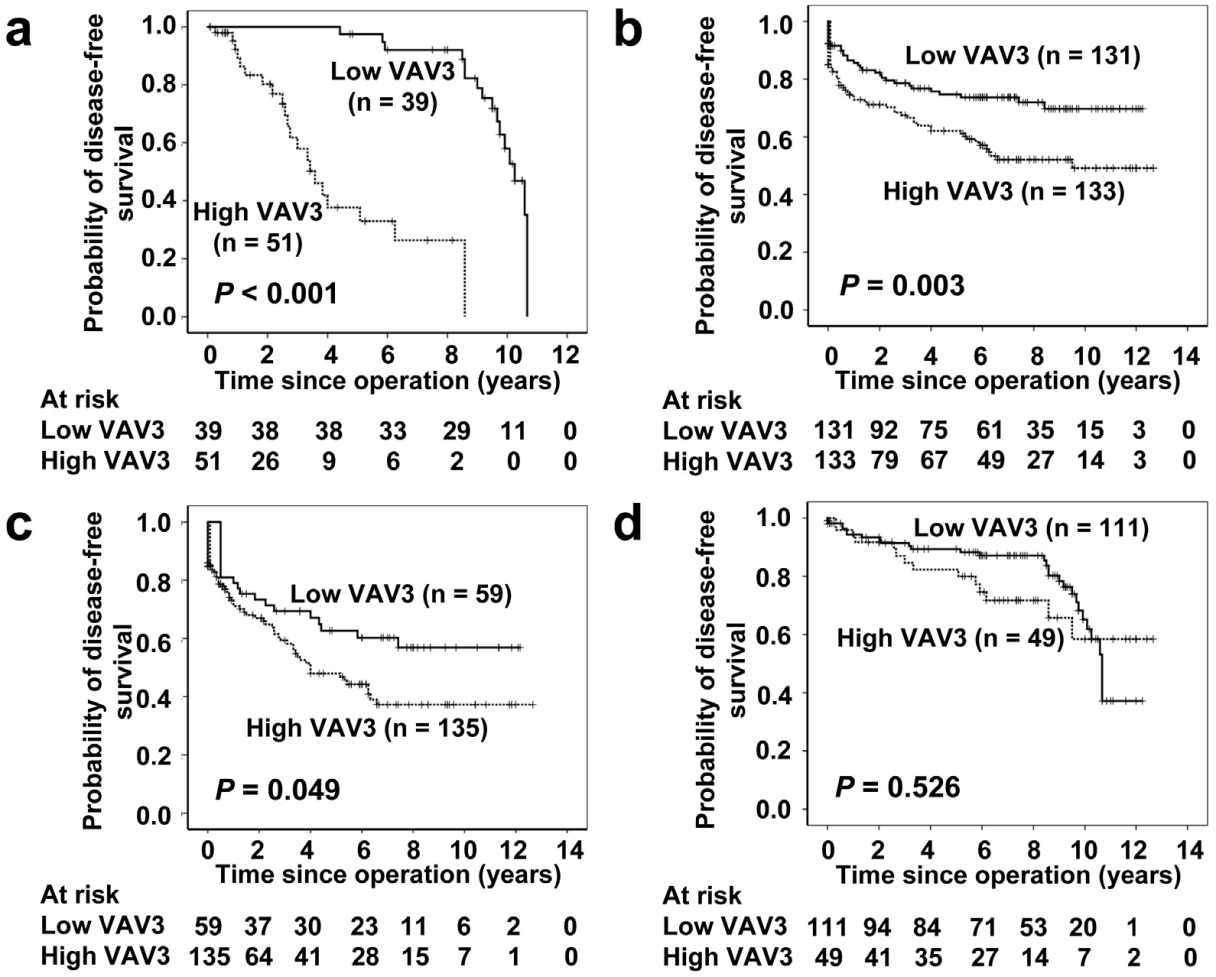
Survival analysis of CRC patients stratified by VAV3 immunoreactivity. Panel a shows the disease-free survival in data set one. Patients with high VAV3 expression had a 10-year disease-free survival rate of 0% compared with 58.0% for patients with low VAV3 expression. Panel b shows the disease-free survival in data set two. Patients with high VAV3 expression had a 10-year disease-free survival rate of 49.2% compared with 69.7% for patients with low VAV3 expression. Panel c shows the disease-free survival in advanced stage CRC (stages III and IV) from the combined data sets one and two. Patients with high VAV3 expression had a 10-year disease-free rate of 37.3% compared with 56.9% for patients with low VAV3 expression. Panel d shows the disease-free survival in early stage CRC (stages I and II) from the combined data sets one and two. Patients with high VAV3 expression had a 10-year disease-free rate of 58.5% compared with 65.1% for patients with low VAV3 expression. All statistical tests were two-sided. Significance level: *P* < 0.05.

Figure 2 shows the correlations of clinical outcomes with VAV3 expression. In data set one, inferior disease-free survival was significantly associated with VAV3 overexpression (*P* < 0.001) (Figure 2a). At 5 years, 36 VAV3-low patients were at risk, and the disease-free survival was 0.974 (95% confidence interval [CI] 0.925 to 1.023); 8 VAV3-high patients were at risk, and the disease-free survival was 0.377 (95% CI 0.191 to 0.563). At 10 years, 11 VAV3-low patients were at risk, and the disease-free survival was 0.580 (95% CI 0.390 to 0.770); 0 VAV3-high patients were at risk, and the disease-free survival was 0 (95% CI 0 to 0).

Table 1 summarizes the univariate analysis of prognostic markers and patient survival in data set one. The nodal status (*P* < 0.001), the stage (*P* < 0.001), and VAV3 overexpression (*P* < 0.001) were significantly correlated with disease-free survival.

The association between VAV3 overexpression and disease-free survival remained significant, even after controlling for other well-known prognostic markers in the multivariate analysis (Table 1). In the multivariate analysis, VAV3 overexpression was prognostically independent (hazard ratio [HR] 16.190, 95% CI 5.083 to 51.565, *P* < 0.001), as were the nodal status (HR 5.512, 95% CI 1.548 to 15.215, *P* = 0.002) and the stage (HR 5.594, 95% CI 1.858 to 16.847, *P* = 0.003).

### Validation of VAV3 overexpression for survival prediction by data set two

In data set two, inferior disease-free survival was significantly associated with VAV3 overexpression (*P* = 0.003) (Figure 2b). At 5 years, 74 VAV3-low patients were at risk, and the disease-free survival was 0.747 (95% CI 0.669 to 0.825); 66 VAV3-high patients were at risk, and the disease-free survival was 0.621 (95% CI 0.535 to 0.707). At 10 years, 15 VAV3-low patients were at risk, and the disease-free survival was 0.697 (95% CI 0.603 to 0.791); 15 VAV3-high patients were at risk, and the disease-free survival was 0.492 (95% CI 0.386 to 0.598).

Table 1 summarizes the univariate analysis of prognostic markers and patient survival in data set two. The depth of invasion (*P* = 0.001), the nodal status (*P* < 0.001), distant metastasis (*P* < 0.001), the stage (*P* < 0.001), perineural invasion (*P* = 0.001), vascular invasion (*P* < 0.001), and VAV3 overexpression (*P* = 0.004) were significantly correlated with disease-free survival.

In the multivariate analysis, VAV3 overexpression was prognostically independent (HR 1.533, 95% CI 1.064 to 2.437, *P* = 0.041), as were the depth of invasion (HR 2.377, 95% CI 1.003 to 5.633, *P* = 0.049) and distant metastasis (HR 18.305, 95% CI 8.292 to 40.410, *P* < 0.001) (Table 1).

### The effect of VAV3 overexpression on the prognosis of advanced stage CRC

The tumor stage is an important prognostic marker of CRC. To determine the effect of VAV3 overexpression on the prognosis of advanced stage CRC (stages III and IV), we used the combined samples of data sets one and two for this analysis. We found that advanced stage CRC concomitant with VAV3 overexpression had a significantly lower 10-year overall survival rate than advanced stage CRC without VAV3 overexpression (Figure 2c, *P* = 0.049), whereas early stage CRC (stages I and II) had a better 10-year overall survival rate, regardless of the VAV3 expression status (Figure 2d, *P* = 0.526).

### The effect of VAV3 overexpression on the prognosis of colon and rectal cancers

CRC can be stratified into colon and rectal cancers according to the anatomical site. To determine the effect of VAV3 overexpression on the prognoses of two groups of colon and rectal cancer patients, we used the combined samples of data sets one and two for this analysis. We found that for both groups of colon and rectal cancer patients, patients with colon and rectal cancers concomitant VAV3 overexpression had a significantly lower 10-year overall survival rate than those with colon and rectal cancers without VAV3 overexpression (Supplementary Fig. S1a and S1b, *P* = 0.002 and 0.021, respectively).

### Effects of VAV3 knockdown on CRC cell growth, spread, and xenograft proliferation

**Figure 3:**
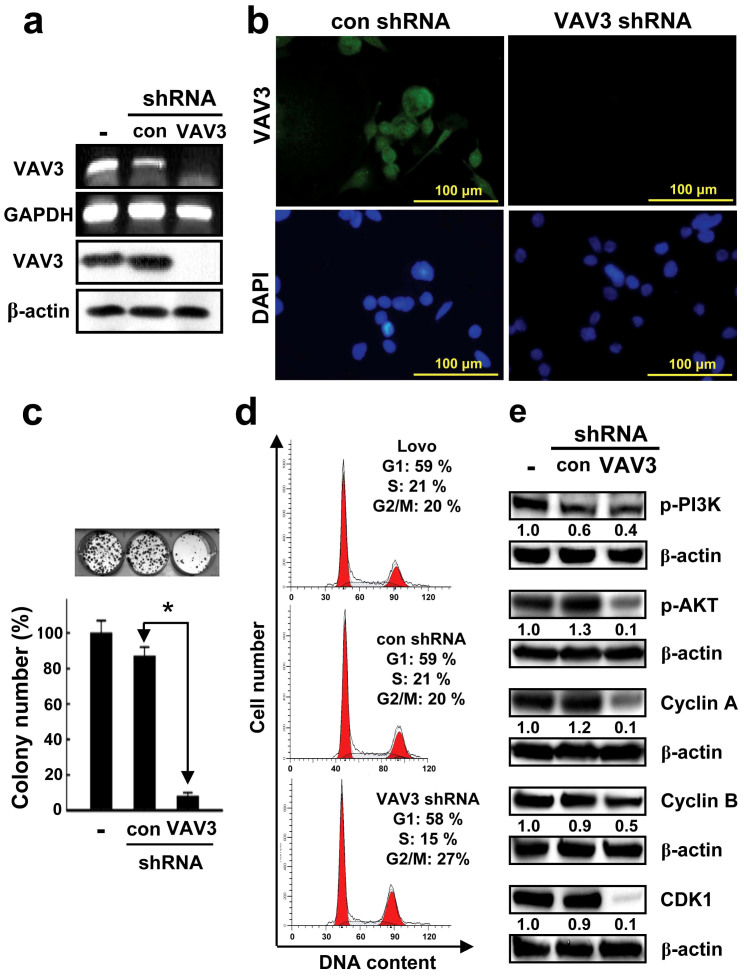
Verification of VAV3 knockdown in LoVo cells, and the effect of stable VAV3 knockdown on LoVo cell growth, cell cycle distribution, and the expression of cell cycle control molecules, phospho-PI3K, and phospho-AKT in LoVo cells. The RT-PCR (a), immunoblotting (a) and immunofluorescence (b) results indicate complete knockdown of VAV3 mRNA and protein expression. The agarose gels in the figure were cropped. The blots in the figure were also cropped, but the polyacrylamide gels were run under the same experimental conditions. (c) Stable VAV3 knockdown results in significantly decreased colony formation. The photomicrographs shown are from one representative experiment performed in triplicate with similar results. The histogram represents the colony numbers (presented as the mean ± standard deviation (SD), *denotes *P* < 0.0001 compared with the control). (d) Stable VAV3 knockdown results in a sustained accumulation of cells in the G2 phase. Cellular distribution (as percentages) in different phases of the cell cycle (G1, S, and G2/M) is presented as the mean ± SD of 3 independent experiments (*denotes *P* < 0.001 compared with the control). (e) Stable VAV3 knockdown decreases the expression of cell cycle control molecules and the levels of phospho-PI3K and phospho-AKT. The typical result from 3 independent experiments is shown. The blots in the figure were cropped, but the polyacrylamide gels were run under the same experimental conditions.

This study also investigated the involvement of VAV3 in the growth and spread of LoVo CRC cells and in vivo tumor growth in nude mice by using shRNA technology to specifically knock down VAV3 (Figure 3a and 3b). Because the efficiency of VAV3 knockdown was similarly and sufficiently high (>95%) in two clones, one clone (clone ID: TRCN000047699) was selected for the following experiments. The number of colonies of LoVo cells infected with lentiviral vectors encoding the VAV3 shRNA construct was significantly lower than of colonies of cells infected with the control (Figure 3c, *P* < 0.0001). To test whether the growth inhibitory effect of VAV3 knockdown was due to disruption of the cell cycle, flow cytometric analysis was conducted to analyze the cell cycle distribution of the VAV3 knockdown and control LoVo cells. The DNA content profile of VAV3 knockdown LoVo cells indicated that the cell cycle was arrested in the G2/M phase (Figure 3d). To identify the molecular mechanisms that govern the VAV3 knockdown-induced G2/M arrest, we assessed the expression of various cyclins and CDKs involved in cell cycle control in the VAV3 knockdown and control LoVo cells. Reduced expression of cyclins A, B, and cyclin-dependent kinases CDK1, all of which are involved in G2 phase regulation, was observed in the VAV3 knockdown LoVo cells (Figure 3e). By contrast, the expression of cyclin D and cyclin-dependent kinases CDK2 and 4, which are involved in G1 phase regulation, remained unchanged (data not shown). These results imply that VAV3 knockdown inhibits cell cycle progression by reducing the levels of cyclin A, cyclin B, and CDK1. Previous studies have shown that the PI3K/AKT signaling pathway is associated with cell proliferation and survival and that VAV3 overexpression leads to PI3K activation^10^,^19^. Here, we also analyzed the influence of VAV3 knockdown on the PI3K/AKT signaling pathway. As shown in Figure 3e, VAV3 knockdown exhibited inhibitory effects on the levels of phospho-PI3K and phospho-AKT. These data suggest that VAV3 knockdown suppresses cell proliferation in part by inhibiting the PI3K/AKT signaling pathway.

To validate the results from LoVo cells, transfection and shRNA technology were also used to overexpress and knockdown VAV3 in FHC and DLD-1 cells. respectively (Supplementary Fig. S2a). The colony formation assay showed that VAV3 up-regulation significantly increased the number of FHC colonies formed compared with the control (*P* = 0.0370), whereas VAV3 knockdown reduced the number of DLD-1 colonies compared with the control (*P* < 0.0001, Supplementary Fig. S2b). Furthermore, VAV3 overexpression and knockdown exhibited stimulatory and inhibitory effects on the levels of phosphor-PI3K, phosphor-AKT, cyclin A, cyclin B, and CDK1 in FHC cells and DLD-1 cells, respectively (Supplementary Fig, S2c). These data also suggest that VAV3 knockdown suppresses cell proliferation in part by inhibiting the PI3K/AKT signaling pathway.

**Figure 4:**
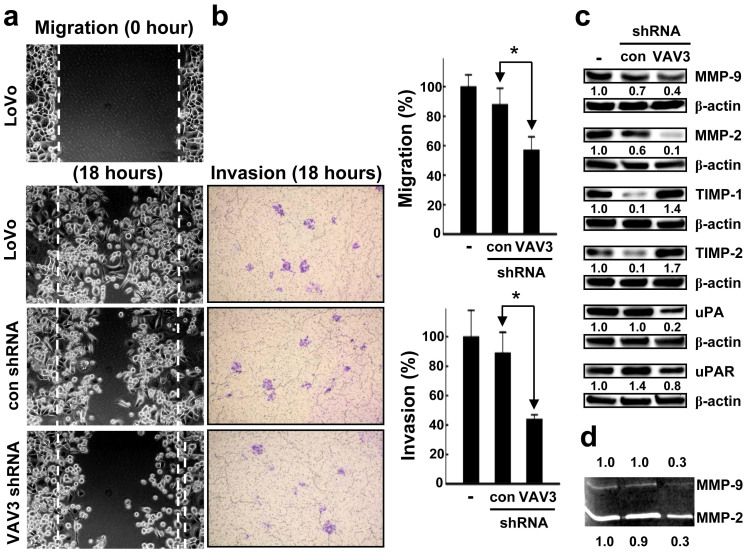
Effect of VAV3 knockdown on LoVo cell spreading and the expression and activities of metastasis-related molecules in LoVo cells. (a) Stable VAV3 knockdown results in a significant decrease in cell migration, as measured by the wound healing assay. The photomicrographs shown are from one representative experiment performed 3 times with similar results. The histogram represents the number of migrated cells (presented as the mean ± SD, *denotes *P* = 0.0205 compared with the control). (b) Stable VAV3 knockdown results in a significant decrease in cell invasion. The number of cells that invaded through the ECMatrix-coated membrane was counted. The photomicrographs shown are from one representative experiment performed in triplicate with similar results. The histogram represents the number of invaded cells (presented as the mean ± SD, *denotes *P* = 0.0255 compared with the control). (c) Stable VAV3 knockdown results in the dysregulated expression of metastasis-related molecules. A typical result from 3 independent experiments is shown. The blots in the figure were cropped, but the polyacrylamide gels were run under the same experimental conditions. (d) Stable VAV3 knockdown results in a significant decrease in the activities of MMP-2 and MMP-9 as measured by gelatin zymography assay. The typical result from 3 independent assays is shown. Gelatin zymography images in the figure were cropped.

To verify the effect of VAV3 knockdown on the migration of the cells, we performed a wound healing assay and observed a significant delay in wound closure in VAV3 knockdown cells compared with control cells (Figure 4a, *P* = 0.0187). In the cell invasion assay, VAV3 knockdown significantly suppressed cell invasion compared with the control (Figure 4a, *P* = 0.0055). MMP-2 and MMP-9 are involved in the breakdown of extracellular matrix in disease processes, such as metastasis. They can be inhibited by specific endogenous tissue inhibitor of metalloproteinases (TIMPs), including TIMP-1 and TIMP-2. Elevated expression level of uPA is found to be correlated with tumor malignancy. It is believed that the tissue degradation following plasminogen activation facilitates tissue invasion and, thus, contributes to metastasis. Immunoblotting was used to analyze the effects of VAV3 knockdown on the expression of the above-mentioned metastasis-related molecules. As shown in Figure 4b, VAV3 knockdown inhibited the expression of MMP-2, MMP-9, uPA, and uPAR. Up-regulated expression of the MMP inhibitors TIMP-1 and TIMP-2 was found after VAV3 knockdown. In addition, the gelatin zymography assay shows that VAV3 knockdown significantly inhibited the activities of MMP-2 and MMP-9 compared with the control (Figure 4c). These results indicate that VAV3 knockdown suppresses cell spreading by dysregulating the expression of metastasis-related molecules.

VAV3 overexpressed FHC cells and VAV3 knockdown DLD-1 cells were used to validate the effects of VAV3 overexpression and knockdown on the migration and invasion of LoVo cells. VAV3 knockdown in DLD-1 cells significantly reduced cell migration and invasion (*P* = 0.0017 and < 0.0001, respectively, Supplementary Fig. S3a). Supporting the role of VAV3 in promoting cell motility, VAV3 overexpression in FHC cells increased invasion and migration (*P* = 0.0091 and 0.0023, respectively, Supplementary Fig. S3a). Furthermore, VAV3 knockdown inhibited the expression of MMP-2 and MMP-9, and up-regulated expression of TIMP-1 and TIMP-2. In contrast, VAV3 overexpression up-regulated the expression of MMP-2 and MMP-9, and inhibited expression of TIMP-1 and TIMP-2 (Supplementary Fig. S3b). Consistent with the MMP-2 and MMP-9 immunobloting, the gelatin zymography assay shows that VAV3 knockdown significantly inhibited the activities of MMP-2 and MMP-9, whereas VAV3 overexpression increased the activities of MMP-2 and MMP-9 (Supplementary Fig. S3c). These results also indicate that VAV3 knockdown suppresses cell spreading by dysregulating the expression of metastasis-related molecules.

Finally, the growth of the LoVo xenografts (Supplementary Fig. S4a and S4b, *P* = 0.0295) was significantly inhibited after VAV3 knockdown compared with the control. Furthermore, VAV3 knockdown significantly suppressed the expression of MMP-2 and MMP-9 in the LoVo xenografts compared with the control (Supplementary Fig. S4c, *P* = 0.0028 and 0.0456, respectively).

## Discussion

CRC is a common malignant neoplasm and also a major cause of cancer-related deaths worldwide. Better knowledge of the molecular mechanisms underlying the development of this deadly malignant neoplasm is, therefore, needed to devise novel prevention and intervention strategies. Specifically, the identification of molecules that are altered during cancer initiation and progression could provide a valuable tool as a prognostic marker or therapeutic target.

VAV proteins regulate many cellular events, including actin remodeling, cell transformation, and oncogenesis^7^,^8^,^20^. For this study, we investigated VAV3 expression in colorectal tissues from CRC patients. As in previous studies of other types of cancer, the results of immunohistochemistry and immunoblotting indicated that VAV3 expression is higher in CRC than in normal tissues and cells^11^,^12^,^13^,^14^,^15^,^16^. Although several studies suggest that VAV3 overexpression could stimulate cancer cell proliferation, the underlying mechanism remains unclear^12^,^14^. Fujikawa et al. showed that VAV3 expression is regulated in a cell cycle-dependent manner. Strikingly, VAV3 is transiently up-regulated in HeLa cells during mitosis^21^. In this study, we explored the role of VAV3 in CRC cell growth by using shRNA technology. Consistent with previous studies, the results indicated that VAV3 knockdown decreased the colony number by inducing cell cycle arrest at the G2/M phase. Furthermore, VAV3 knockdown could inhibit the expression of G2/M phase-related molecules by inhibiting the PI3K-AKT signaling pathway. These findings might partly explain the underlying mechanism of the association between VAV3 overexpression and tumor cell proliferation.

Our study demonstrates that VAV3 overexpression in CRC tissues is closely correlated with tumor invasion and metastasis. The mechanism by which VAV3 exerts its invasive and metastatic activity remains unclear. Previous studies have suggested that invasion-promoting membrane-type MMPs (MT-MMPs) are directly linked to tumorigenesis^22^. The Rho family GTPases can also control several molecular events that lead to a more motile cellular phenotype, including MT-MMP expression^23^,^24^. Bartolomé et al. demonstrated that the up-regulation of MT-MMP expression by CXCL12, a mechanism that contributes to melanoma cell invasion, is blocked by knocking down VAV1 and VAV2 expression^25^. Our results indicated that VAV3 knockdown inhibited the migration and invasion of CRC cells. Furthermore, mechanistic studies revealed that VAV3 knockdown could dysregulate the expression and activities of metastasis-related molecules by inhibiting the PI3K-AKT signaling pathway in both CRC cells and xenografts. These findings may partly account for the association of VAV3 overexpression with tumor invasion and metastasis.

Although it is generally accepted that surgical resection is the most powerful tool for improving the prognosis when CRC is diagnosed early, determining the necessity for an intensive follow-up and adjuvant therapy by predicting recurrence and metastases remains important^26^,^27^,^28^. Studies related to the prognostic value of VAV proteins are scarce. Fernandez-Zapico et al. provided evidence of a strong association between VAV1 overexpression and a poor survival rate in pancreatic cancer patients^11^. Lin et al. showed that VAV3 serves as a prognostic marker for the recurrence of early stage prostate cancer following prostatectomy^15^. Our previous work demonstrated that VAV3 overexpression could be a useful marker for predicting the outcome of gastric cancer patients^16^. In the present study, the clinicopathologic analysis revealed that VAV3 overexpression was significantly correlated with the depth of invasion, the nodal status, distant metastasis, and the tumor stage, and patients who had CRC with high VAV3 expression had a poorer prognosis for disease-free survival than those of the low-expression group. In addition, it is well known that the tumor stage is an important prognostic marker of CRC and that the prognosis of advanced CRC is poor^29^. In this study, we also investigated the association between high VAV3 expression and the prognosis of advanced CRC. The results revealed that patients with advanced stage CRC with VAV3 overexpression had poorer survival rates than those without VAV3 overexpression and should be followed-up carefully. This is the first study to show that VAV3 overexpression can be an independent prognostic marker for CRC following surgical resection.

## Methods

### Study subjects

Paraffin-embedded specimens of CRC tissues from 103 consecutive patients who underwent surgical resection of CRC at Chi Mei Medical Center from 2000 to 2003 were retrospectively studied. Among the 103 patients with CRC, follow-up information was available for 90 patients (data set one). The expression of VAV3 was validated using an independent cohort of 304 consecutive patients who underwent surgical resection of CRC at Taipei Medical University Wan Fang Hospital from 1999 to 2006. Among the 304 patients with CRC, follow-up information was available for 264 patients (data set two). In all surgical specimens, the tumor tissue was obtained from grossly visible tumor lesion, and non-tumor tissue was obtained from grossly normal colorectal tissue in the same specimen away from the tumor lesion. Both paired tumor tissue and non-tumor tissue were taken from the surgical specimen by pathologists following a standard protocol in the department of pathology in both hospitals. In each case, the representative paired tumor and non-tumor paraffin sections were selected for immunohistochemical assay under the microscope by a pathologist (C.-L. Fang) to confirm the presence of tumor tissue or not. By microscopic examination, the tumor component must be predominant, and excess more than 50% of the area of the whole representative tumor section. In this study, all patients were treated according to the standard practice guideline for CRC in both hospitals. In this guideline, all cases of stage IV CRC with distant metastasis were resectable after a series of clinical evaluation. For example, in stage IV rectal cancer patients with liver and/or lung metastasis, adjuvant chemotherapy was applied after transabdominal resection of rectal tumor with removal of regional lymph nodes, and staged or synchronous resection of metastatic lesions. For stage IV colon cancer patients with liver and/or lung metastasis, adjuvant chemotherapy was given after colectomy with staged or synchronous liver and or lung resection. Thus, we can obtain the surgical specimens including tumor and non-tumor tissues from these advanced patients. None of these patients received preoperative chemotherapy and/or radiotherapy. The clinicopathologic parameters of CRC were determined based on the American Joint Committee on Cancer classification. The clinical outcome endpoint was disease-free survival. The follow-up duration for disease-free survival was defined as the period between the operation date and the date of relapse. The final follow-up date for the cases was August 18, 2013. In data set one, retrospective power analysis was conducted to determine the association of VAV3 expression and disease-free survival. It showed that the study had approximately 97% power to detect a significant difference (effect size = 0.51) from the observed data, assuming a significance level of α = 5%. The institutional review boards of both hospitals approved this study, and written ethical consent was obtained from all patients. Tumor and non-tumor pairs of colorectal tissues were analyzed for VAV3 expression.

### Immunohistochemical analysis

Paraffin-embedded sections were stained with primary antibodies at 4°C overnight. A standard peroxidase-conjugated streptavidin-biotin method was used to detect the immunoreactivity (Dako REAL EnVision Detection System; Dako, Carpinteria, CA, USA). Breast cancer tissue was used as a positive control for VAV3. Following the method adopted in a previous study, VAV3 immunoreactivity was assessed semiquantitatively and scored as follows: 0, no staining; 1, weak and focal staining in <25% of the tissue; 2, moderate staining in 25%–50% of the tissue; and 3, strong staining in >50% of the tissue^14^. Sections with a score of 0 or 1 exhibited low expression of VAV3, and those with a score of 2 or 3 were defined as exhibiting high expression or overexpression of VAV3. Clinical data collection and immunohistochemical analysis were independently performed in an investigator-blinded study.

### Cell culture

Human normal (FHC) and CRC (LoVo and DLD-1) cell lines were obtained from the American Type Culture Collection (Manassas, VA, USA). FHC cells were established from normal fetal colonic mucosa. Cell lines were authenticated by the ATCC cell biology program and were not passaged for longer than six months before thawing and using the original frozen stocks or purchasing a new cell aliquot from the ATCC. Cells were cultured in DMEM/F12 (FHC), F-12K (LoVo) or RPMI-1640 (DLD-1) media supplemented with 10% fetal bovine serum (FBS), 100 units/mL penicillin G, 100 μg/mL streptomycin sulfate, and 250 ng/mL amphotericin B. For the culture of FHC cells, 0.005 mg/mL insulin, 0.005 mg/mL transferrin, 100 ng/mL hydrocortisone, and extra 10 mM HEPES were added.

### shRNA treatment

For shRNA treatment, LoVo and DLD-1 cells were infected with lentiviral vectors (two VAV3-shRNA constructs, clone IDs: TRCN0000047698, TRCN0000047699, and one control, clone ID: pLKO_TRC025, purchased from the National RNAi Core Facility, Taiwan) and stable clones resistant to puromycin (Santa Cruz, Santa Cruz, CA, USA) were selected. Reverse-transcription polymerase chain reaction (RT-PCR), immunoblotting and immunofluorescence were performed to evaluate the effects of shRNA treatment.

### Transfection

FHC cells were transfected with Human VAV3 cDNA ORF and empty vectors (OriGene, Rockville, MD, USA) and stable clones resistant to G418 (St. Louis, MO, USA) were selected. RT-PCR and immunoblotting were performed to evaluate the efficiency of transfection.

### Colony formation assay

The cell proliferation was analyzed by a colony formation assay, as detailed in the Supplementary methods (available online).

### Flow cytometric analysis

The cellular DNA content was determined by flow cytometric analysis of propidium iodide (PI)-labeled cells, as described in our previous study^17^.

### Wound healing assay

The cell migratory ability was examined by a wound healing assay, as described in detail in the Supplementary methods (available online).

### In vitro invasion assay

The cell invasion capability was examined using a Cell Invasion Assay Kit obtained from Millipore (Temecula, CA, USA), as detailed in the Supplementary methods (available online).

### Gelatin zymography assay

The activities of matrix metalloproteinase-2 (MMP-2) and MMP-9 in the medium were measured by gelatin zymography protease assays, as previously detailed^18^.

### Tumor cell inoculation

Animals were divided into 2 groups (4 mice/group) for tumor cell inoculation. The VAV3 knockdown and control LoVo cells (5 × 10^6^) were mixed in a 200 μL Matrix gel and injected subcutaneously on the right hind flank. Tumor volume was measured every other day after inoculation. The mice were photographed and sacrificed 5 weeks after inoculation. The tumors were then removed and weighed.

### Statistical analysis

All of the data were analyzed using the SPSS software version 17.0 (SPSS, Inc., Chicago, IL, USA). All statistical tests were two-sided, and a *P* value of < 0.05 was considered to be significant. All of these methods and statistical analyses were described in detail in the Supplementary methods (available online).

## Author Contributions

Y.-H.U. participated in study conception and design, collected the patient tissues, and was involved in drafting the manuscript. C.-L.F. collected the patient tissues, and revised the manuscript for important intellectual content. Y.-C.H. analyzed and interpreted the data, and revised the manuscript for important intellectual content. P.-C.S., K.-S.W. and S.-T.H. performed the experiments. H.-L.Y. analyzed and interpreted the data. L.-H.W. participated in study conception and design. K.-Y.L. participated in study conception and design, analyzed and interpreted the data, and was involved in drafting the manuscript. All authors read and approved the final manuscript.

## Supplementary Material

Supplementary Information Supplementary Information

## Figures and Tables

**Table 1:** Univariate and multivariate Cox regression analyses of prognostic markers and survival in data sets one and two

	Univariate		Multivariate	
Variable	HR (95% CI)	P*	HR (95% CI)	P*
Data set one				
VAV3 Low expression vs. High expression	15.237 (5.506 to 42.162)	<0.001	16.190 (5.083 to 51.565)	<0.001
Depth of invasion T1 + T2 vs. T3 + T4	1.149 (0.537 to 2.457)	0.721	0.416 (0.165 to 1.052)	0.064
Nodal status N0 vs. N1 + N2 + N3	7.063 (3.012 to 19.236)	<0.001	5.512 (1.548 to 15.215)	0.002
Stage I + II vs. III + IV	8.073 (3.095 to 21.057)	<0.001	5.594 (1.858 to 16.847)	0.003
Perineural invasion Absence vs. Presence	1.384 (0.567 to 3.380)	0.476	1.042 (0.323 to 3.367)	0.945
Vascular invasion Absence vs. Presence	1.769 (0.871 to 3.594)	0.115	0.935 (0.348 to 2.508)	0.893
Data set two				
VAV3 Low expression vs. High expression	1.871 (1.218 to 2.873)	0.004	1.533 (1.064 to 2.437)	0.041
Depth of invasion T1 + T2 vs. T3 + T4	3.974 (1.735 to 9.102)	0.001	2.377 (1.003 to 5.633)	0.049
Nodal status N0 vs. N1 + N2 + N3	2.277 (1.462 to 3.546)	<0.001	1.251 (0.502 to 3.114)	0.631
Distant metastasis Absence vs. Presence	22.037 (11.807 to 241.129)	<0.001	18.305 (8.292 to 40.410)	<0.001
Stage I + II vs. III + IV	3.382 (2.072 to 5.522)	<0.001	1.119 (0.399 to 3.137)	0.831
Perineural invasion Absence vs. Presence	2.143 (1.380 to 3.326)	0.001	1.233 (0.760 to 2.001)	0.397
Vascular invasion Absence vs. Presence	2.937 (1.849 to 64.667)	<0.001	1.674 (1.000 to 2.800)	0.050

*All statistical tests were two-sided. Significance level: *P* < 0.05.
