# Supplementary material for: VAV3 Oncogene Expression in Colorectal Cancer: Clinical Aspects and Functional Characterization
Source: Sci Rep. 2015 Mar 20;5:9360. doi: 10.1038/srep09360 (PMC4366846; doi:10.1038/srep09360)
Supplement: Supplementary Information [file srep09360-s1.pdf]

Supplementary information: Supplementary methods, Supplementary tables, Supplementary figure legends and Supplementary figures

"*VAV3* Oncogene Expression in Colorectal Cancer: Clinical Aspects and Functional Characterization" by Yih-Huei Uen, Chia-Lang Fang, You-Cheng Hseu, Pei-Chun Shen, Hsin-Ling Yang, Kuo-Shan Wen, Shih-Ting Hung, Lu-Hai Wang & Kai-Yuan Lin

Supplementary methods

Antibodies. The antibody against VAV3 was purchased from Millipore (Temecula, CA, USA). The antibody against cyclin B was purchased from Life Technologies (Grand Island, NY, USA). The antibody against CDK1 was obtained from Abgent (San Diego, CA, USA). The antibodies against CDK2, MMP-2, TIMP-1, TIMP-2, uPA, uPAR, and Ki67 were from Santa Cruz. The antibodies against p-PI3K, p-Akt, cyclin A, cyclin D, CDK4, and MMP-9 were purchased from Cell Signaling Technology (Danvers, MA, USA). The antibody against β-actin was obtained from Sigma.

Immunoblotting. Total tissue and cell lysates were extracted with RIPA Buffer (Pierce, Rockford, IL, USA). Denatured protein samples were subjected to 10% SDS-PAGE and transferred to nitrocellulose membranes. Blocked blots were incubated at 4°C overnight with primary antibodies. β-Actin was used as an internal control for equal protein loading. After incubation with secondary antibodies conjugated with peroxidase (Sigma), enhanced chemiluminescence reagents (Pierce) were used to visualize the targeted proteins. All experiments were conducted 3 times independently.

RNA extraction and RT-PCR analysis. Total RNA from VAV3 knockdown and control LoVo cells was extracted using TRIzol reagent according to the manufacturer's protocol

(Sigma). After chloroform separation, the aqueous phase was collected, and RNA was precipitated by adding ice-cold isopropanol. After washing once with 75% ethanol, the pellets were air-dried to determine the RNA concentrations. Complementary DNA (cDNA) was synthesized from total RNA using a QuantiTect Reverse Transcription Kit (Qiagen, Valencia, CA, USA) following the instructions of the manufacturer. Total RNA was mixed with Genomic DNA Wipeout Buffer and incubated at 42 °C for 2 min. Quantiscript RT Buffer, RT Primer Mix, and Quantiscript Reverse Transcriptase were added and incubated at 42°C for an additional 30 min. Synthesized cDNA and the following primers were used to detect VAV3 expression: forward primer 5'-CGA ACA CCT ATA GCA GCC GC-3' and reverse primer 5'-GGT GGA GAA GTC TAT GAG GAC-3'. The annealing temperature for VAV3 was 54.4°C. The RT-PCR products were separated by 0.5× TBE agarose gel electrophoresis and were visualized by staining with 0.5 μg/mL ethidium bromide. All experiments were performed in triplicate.

Immunofluorescence. The VAV3 knockdown and control LoVo cells were grown in chamber slides (Millipore). Cells were fixed with acetone at -20°C for 5 min. After blocking, fixed cells were incubated at 4°C overnight with an anti-VAV3 antibody (1:50 dilution). Goat anti-rabbit IgG conjugated to fluorescence isothiocyanate (Life Technologies, Grand Island, New York, USA) at a concentration of 0.25 μg/mL was used as the secondary antibody.

After counterstaining with DAPI, the slides were mounted and photographed (200× magnification).

Colony formation assay. Five hundred cells were seeded into 6-well plates and cultured for 12 days. Individual colonies (> 50 cells/colony) were fixed, stained using a solution of 1% crystal violet in methanol, and counted. The assay was conducted three 3 times, and the results were presented as the mean ± SD.

Wound healing assay. For the wound healing assay, cells (5×10^5) were seeded into a 12-well culture dish and grown to a nearly confluent monolayer. The monolayers were carefully scratched using a 200 μL pipette tip. Cellular debris was removed by washing with 1× PBS, and the cells were then incubated for 18 h. The cultures were photographed (100× magnification) at 0 and 18 h to monitor the migration of cells to the wounded area, and the number of migrated cells was quantified using Image J software. All experiments were performed in triplicate, and the results were presented as the mean ± SD.

In vitro invasion assay. The cell invasion capability was examined using a Cell Invasion Assay Kit (Millipore), following the manufacturer’s instructions. Complete media were first added to 24-well plates. The cells (2×10^5) in serum-free media were added to

ECMatrix-layered cell culture inserts (containing 8 μm pore size polycarbonate membranes) and cultured for 24 h. Before staining, the cells on the upper surface were removed. Inserts were then dipped in the Staining Solution to stain invaded cells on the lower surface of the membranes. The cultures were photographed (100× magnification), and the number of invaded cells was counted. The assay was conducted 3 times independently, and the results were presented as the mean ± SD.

Animals. Eight 5-week-old male athymic nude mice (BALB/c *nu*/*nu*) were purchased from the National Laboratory Animal Center, Taiwan. The animals were caged in a specifically designed pathogen-free isolation facility with a 12/12-h light-dark cycle and were provided with rodent chow and water ad libitum. All experiments were conducted in accordance with the guidelines of the China Medical University Animal Ethics Research Board.

Statistical analysis. The difference in VAV3 expression between tumor and non-tumor tissues in the same patient was analyzed using a paired *t* test. The differences in growth, migration, and invasion between VAV3 knockdown and control cells were also analyzed using a Student's *t* tests. We examined some clinicopathologic parameters, including age, gender, depth of invasion, nodal status, distant metastasis, stage, degree of differentiation, perineural invasion, and vascular invasion. The correlation between VAV3 expression and

the clinicopathologic parameters was examined using the χ^2 test. Survival analysis was first performed using the Kaplan-Meier method, and statistical significance was then determined using the log-rank test. The Cox proportional hazards model was used for univariate and multivariate analyses to determine the relative prognostic effect of VAV3 and other potential prognostic markers. The assumption of proportionality of hazards functions was assessed using graphical methods. All data were analyzed using SPSS version 17.0 (SPSS, Inc., Chicago, IL, USA). All statistic tests were two-sided, and a *P* value of < 0.05 was considered significant.

Table S1. Clinical characteristics of CRC patients according to high or low VAV3 expression in data sets one and two

Characteristics	Patients with low VAV3 expression (%)	Patients with high VAV3 expression (%)	*P**
Data set one (n = 90)	n = 39	n = 51	
Age (mean ± SD)	63.8 ± 13.5	59.7 ± 14.1	0.1691
Gender			0.9885
Male	23 (59)	30 (59)	
Female	16 (41)	21 (41)	
Depth of invasion			0.0266
T1 + T2	13 (33)	7 (14)	
T3 + T4	26 (67)	44 (86)	
Nodal status			< 0.0001
N0	31 (79)	13 (25)	
N1 + N2 + N3	8 (21)	38 (75)	
Stage			< 0.0001
I + II	29 (74)	15 (29)	
III + IV	10 (26)	36 (71)	

Perineural invasion			0.9435
Absence	35 (90)	46 (90)	
Presence	4 (10)	5 (10)	
Vascular invasion			0.2444
Absence	33 (85)	38 (75)	
Presence	6 (15)	13 (25)	
Data set two (n = 264)	n = 131	n = 133	
Age (mean ± SD)	69.6 ± 11.0	66.7 ± 14.6	0.0658
Gender			0.8842
Male	77 (59)	77 (58)	
Female	54 (41)	56 (42)	
Depth of invasion			0.0259
T1 + T2	33 (25)	19 (14)	
T3 + T4	98 (75)	114 (86)	
Nodal status			< 0.0001
N0	115 (88)	85 (64)	
N1 + N2 + N3	16 (12)	48 (36)	

Distant metastasis			0.0354
M0	119 (91)	109 (82)	
M1	12 (9)	24 (18)	
Stage			< 0.0001
I + II	80 (61)	36 (27)	
III + IV	51 (39)	97 (73)	
Perineural invasion			0.5093
Absence	100 (76)	106 (80)	
Presence	31 (24)	27 (20)	
Vascular invasion			0.0654
Absence	70 (53)	56 (42)	
Presence	61 (47)	77 (58)	

* All statistical tests were two-sided. Significance level: $P < 0.05$.

Supplementary figure legends

Figure S1. Survival analysis of colon and rectal cancer patients stratified by VAV3 immunoreactivity. **Panel a** shows the disease-free survival in colon cancer patients. Patients with high VAV3 expression had a 10-year disease-free survival rate of 40.8% compared with 66.3% for patients with low VAV3 expression. **Panel b** shows the disease-free survival in rectal cancer patients. Patients with high VAV3 expression had a 10-year disease-free survival rate of 48.2% compared with 51.4% for patients with low VAV3 expression.

Figure S2. Verification of VAV3 manupulation in FHC and DLD-1 cells, and the effect of stable VAV3 manipulation on cell growth, cell cycle distribution, and the expression of cell cycle control molecules, phospho-PI3K, and phospho-AKT in the cells. The RT-PCR and immunoblotting results **(a)** indicate VAV3 was efficiently overexpressed by transfection and knockdown by shRNA treatment. The agarose gels in the figure were cropped. The blots in the figure were also cropped, but the polyacrylamide gels were run under the same experimental conditions. **(b)** Stable VAV3 overexpression and knockdown results in significantly increased and decreased colony formation, respectively. The photomicrographs shown are from one representative experiment performed in triplicate with similar results. The histogram represents the colony numbers (presented as the mean ± standard deviation (SD), *denotes $P < 0.0001$ compared with the control). (**c)** Stable VAV3 overexpression and knockdown significantly increased and decreased the expression of

cell cycle control molecules and the levels of phospho-PI3K and phospho-AKT, respectively. The typical result from 3 independent experiments is shown. The blots in the figure were cropped, but the polyacrylamide gels were run under the same experimental conditions.

Figure S3. Effect of VAV3 manipulation in FHC and DLD-1 cells on cell spreading and the expression and activities of metastasis-related molecules. **(a)** Stable VAV3 overexpression and knockdown significantly increased and decreased cell migration and invasion. The photomicrographs shown are from one representative experiment performed 3 times with similar results. The histogram represents the number of migrated cells (presented as the mean ± SD, *denotes P = 0.0205 compared with the control). **(b)** Stable VAV3 manipulation results in the dysregulated expression of metastasis-related molecules. A typical result from 3 independent experiments is shown. The blots in the figure were cropped, but the polyacrylamide gels were run under the same experimental conditions. **(c)** Stable VAV3 overexpression and knockdown significantly increased and decreased the activities of MMP-2 and MMP-9, respectively. The typical result from 3 independent assays is shown. Gelatin zymography images in the figure were cropped.

Figure S4. Effect of stable VAV3 knockdown on in vivo tumor growth in nude mice. **(a)** **Upper panel** There was no difference in body weight between nude mice in two groups. **Lower panel** Stable VAV3 knockdown results in a time-dependent decrease of the volume

of the LoVo xenograft. The results are presented as the mean ± SD (n = 4). **(b)** Stable VAV3 knockdown results in the significant inhibition of LoVo xenograft proliferation. The histogram represents the results of the average tumor weight (presented as the mean ± SD, *denotes P = 0.0295 compared with the control). **(c)** Stable VAV3 knockdown results in the significant inhibition of MMP-2 and MMP-9 expression in LoVo xenografts. The typical result from 3 independent experiments is shown. The blots in the figure were cropped, but the polyacrylamide gels were run under the same experimental conditions. The histogram represents the results of the average protein levels (presented as the mean ± SD, *denotes P = 0.0028 and 0.0456, respectively, compared with the control).

a

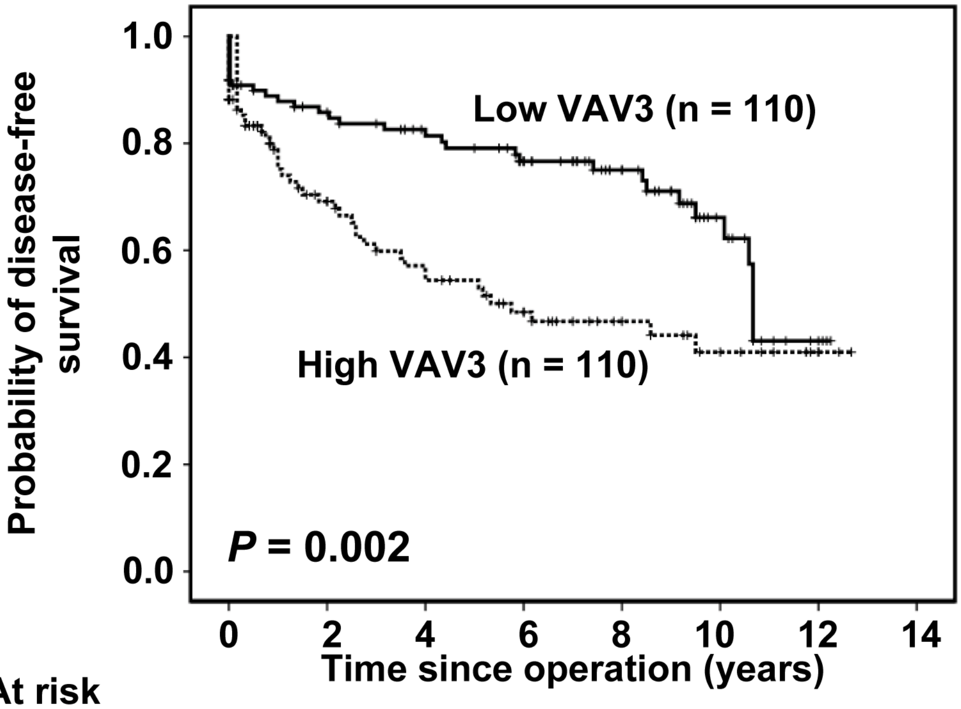

At risk								
Low VAV3	110	81	70	56	39	17	3	0
High VAV3	110	53	40	28	18	10	2	0

b

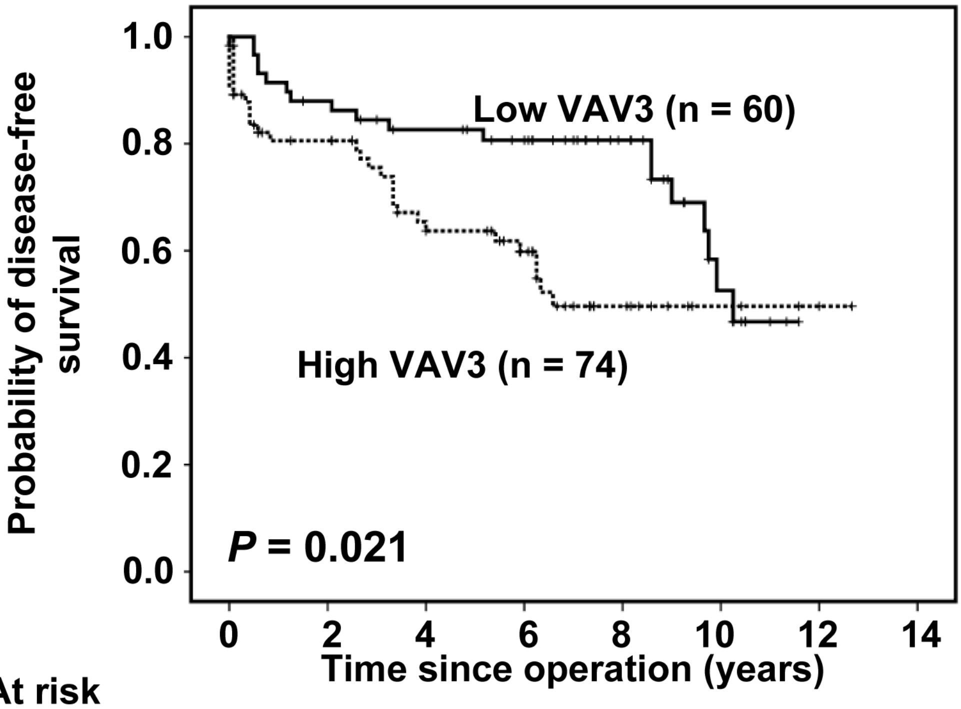

At risk								
Low VAV3	60	50	44	38	25	9	0	0
High VAV3	74	52	36	27	11	4	1	0

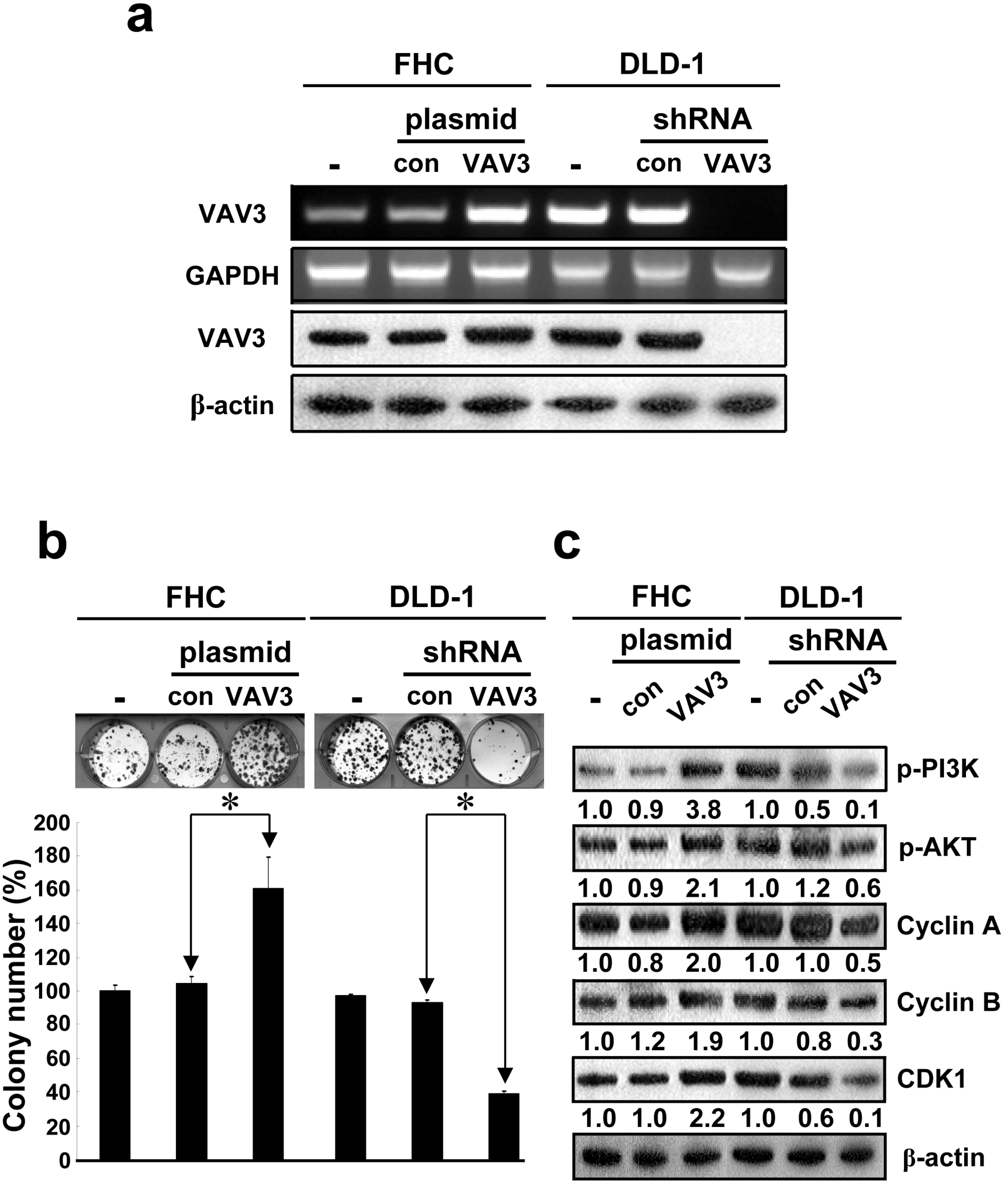

a
FHC
DLD-1
plasmid
shRNA
-
con
VAV3
-
con
VAV3
VAV3
GAPDH
VAV3
β-actin
b
FHC
DLD-1
plasmid
shRNA
-
con
VAV3
-
con
VAV3
*
*
Colony number (%)
200
180
160
140
120
100
80
60
40
20
0
c
FHC
DLD-1
plasmid
shRNA
-
con
VAV3
-
con
VAV3
p-PI3K
1.0 0.9 3.8 1.0 0.5 0.1
p-AKT
1.0 0.9 2.1 1.0 1.2 0.6
Cyclin A
1.0 0.8 2.0 1.0 1.0 0.5
Cyclin B
1.0 1.2 1.9 1.0 0.8 0.3
CDK1
1.0 1.0 2.2 1.0 0.6 0.1
β-actin

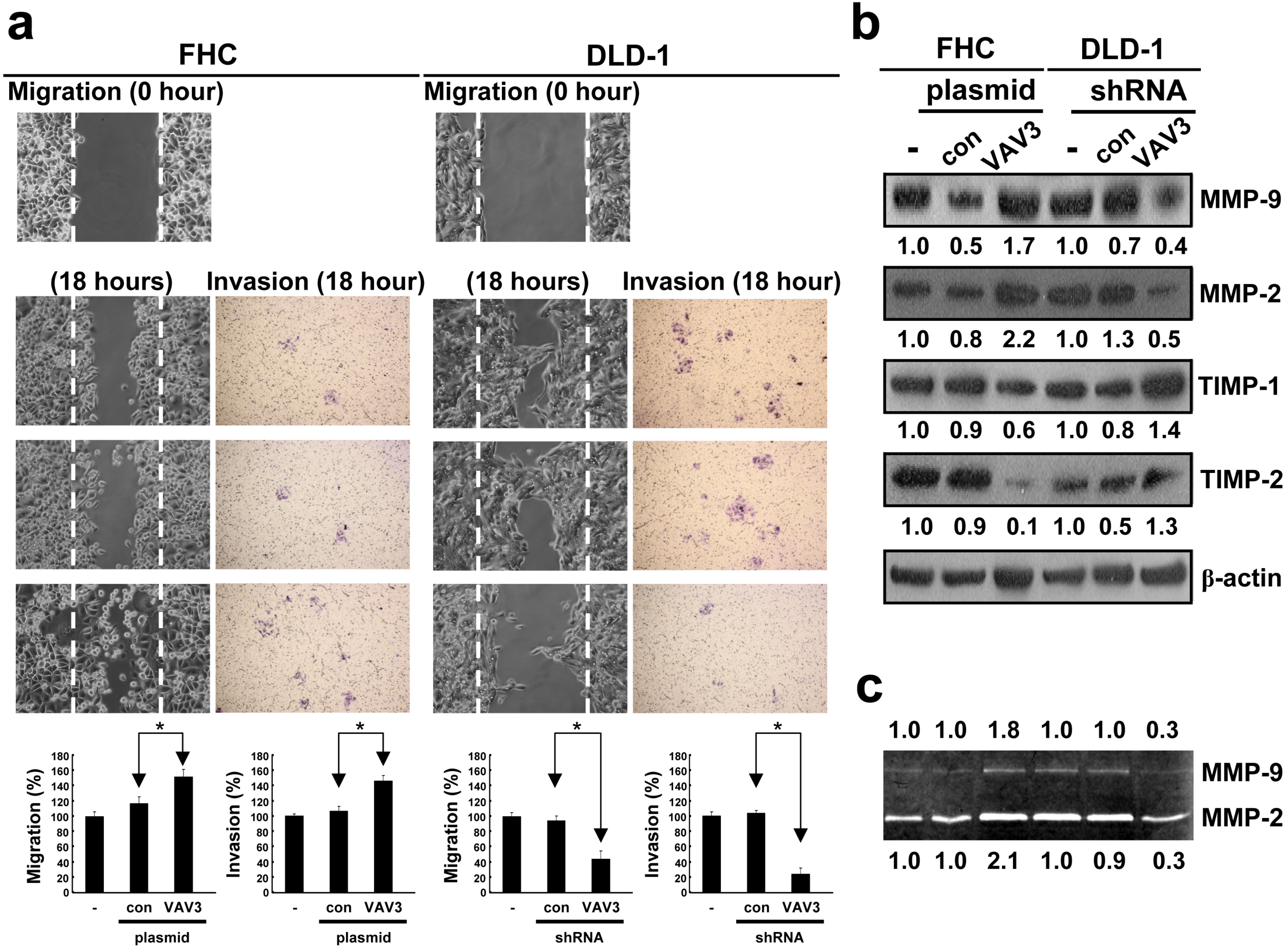
a
FHC
DLD-1
Migration (0 hour)
Migration (0 hour)
(18 hours)
Invasion (18 hour)
(18 hours)
Invasion (18 hour)
Migration (%)
Invasion (%)
- con VAV3
plasmid
shRNA
*
b
FHC
DLD-1
plasmid
shRNA
- con VAV3 - con VAV3
MMP-9
1.0 0.5 1.7 1.0 0.7 0.4
MMP-2
1.0 0.8 2.2 1.0 1.3 0.5
TIMP-1
1.0 0.9 0.6 1.0 0.8 1.4
TIMP-2
1.0 0.9 0.1 1.0 0.5 1.3
β-actin
c
1.0 1.0 1.8 1.0 1.0 0.3
MMP-9
MMP-2
1.0 1.0 2.1 1.0 0.9 0.3

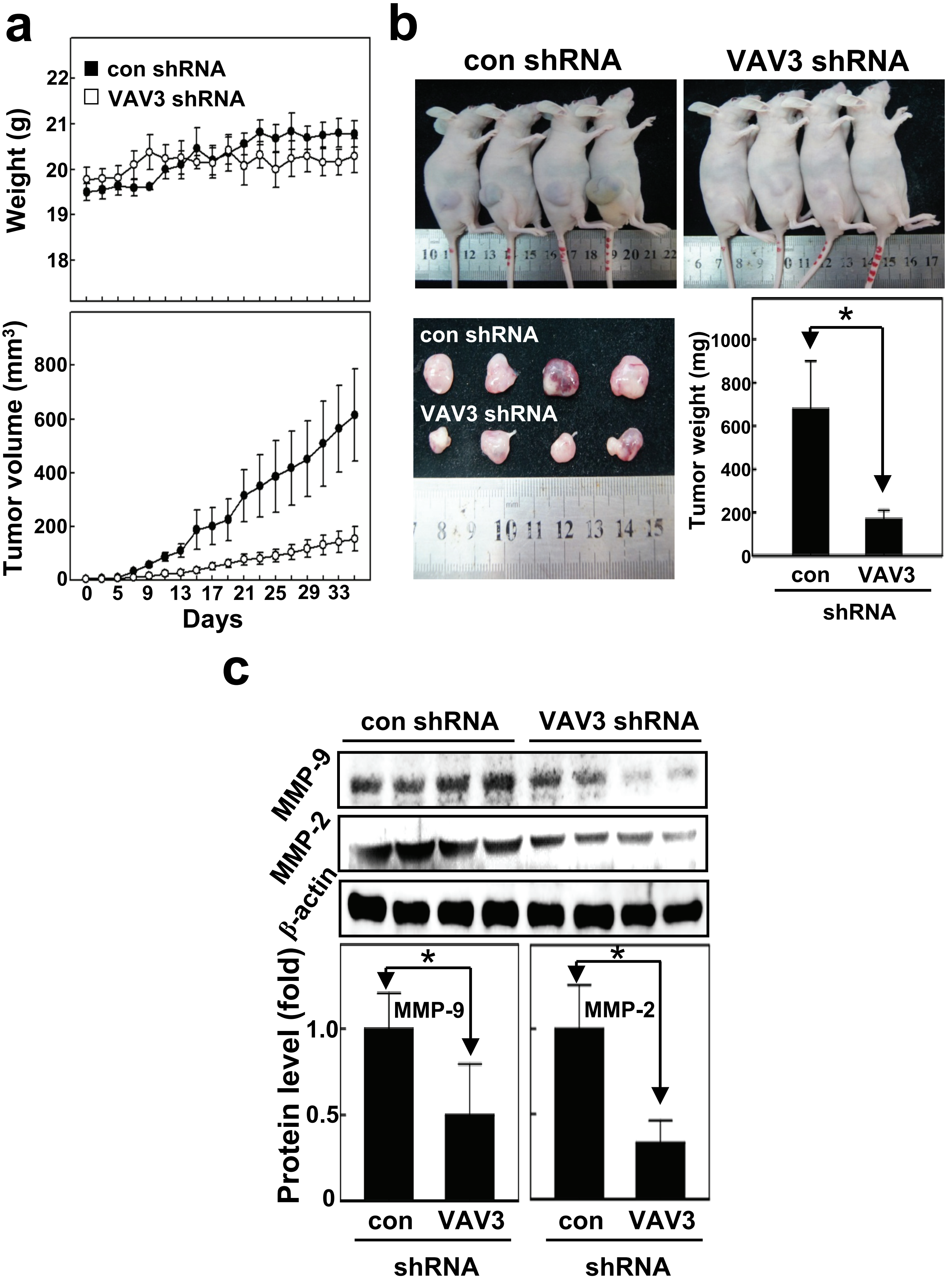
a
con shRNA
VAV3 shRNA
Weight (g)
22
21
20
19
18
Tumor volume (mm³)
800
600
400
200
0
0 5 9 13 17 21 25 29 33
Days
b
con shRNA
VAV3 shRNA
con shRNA
VAV3 shRNA
Tumor weight (mg)
1000
800
600
400
200
0
*
con VAV3
shRNA
c
con shRNA
VAV3 shRNA
MMP-9
MMP-2
β-actin
Protein level (fold)
1.0
0.5
0
*
MMP-9
MMP-2
con VAV3
shRNA
con VAV3
shRNA
